# The Fracture Characteristic of Three Collinear Cracks under True Triaxial Compression

**DOI:** 10.1155/2014/459025

**Published:** 2014-03-20

**Authors:** Jianjun Liu, Zheming Zhu, Bo Wang

**Affiliations:** ^1^State Key Laboratory of Oil and Gas Reservoir Geology and Exploitation, School of Civil Engineering and Architecture, Southwest Petroleum University, Chengdu 610500, China; ^2^Department of Engineering Mechanics, Sichuan University, Chengdu 610065, China

## Abstract

The mechanical behavior of multicracks under compression has become a very important project in the field of fracture mechanics and rock mechanics. In this paper, experimental and numerical studies on the fracture property of three collinear cracks under compression were implemented. The specimens were a square concrete plate, and the cracks were made by a very thin film. The tests were conducted by using true triaxial loading device. In the numerical study, the Abaqus code was employed. The effect of crack orientation and the confining stress on cracked specimen compressive strength were investigated. The results show that the critical stresses of cracked specimens change with crack inclination angles, and, as the angle is 45°, the critical stress is the lowest; the critical stresses increase with the confining stresses.

## 1. Introduction

Geological faults may not be continuous, but discrete, and they could be lined up which is called collinear discontinuous faults. How to predict the collinear discontinuous fault stability is a significant subject because the main manifestations of faulting are earthquakes, which could cause large disasters; thus the understanding of the factors that control fault ruptures is a major task for seismologists and other earth scientists. There are many factors which could affect fault stability, for example, the stress state, the fault orientation, the fault friction, and the target rock strength.

In order to investigate the fracture mechanism of cracked rock, in 1963, Brace and Bombolakis [1] conducted the compression tests of the rock samples with a centralized inclination crack, and they found that under uniaxial and biaxial compression, the inclined crack did not propagate straightly, but deviated in an angle from the original crack plane, which is currently called “wing-crack,” and the angle was about 70°. As the loading continued to increase, the crack finally tended to parallel the major principal stress. In 1965, similar tests were conducted by Hoek and Bieniawski [2], and they obtained the similar results. Latterly, testing results [3–8] showed that the wing-crack is a common phenomenon as a brittle material under compression. In the experiment of gypsum specimens under uniaxial and biaxial compression, Bobet [9, 10] also observed wing-cracks initiating from crack tips. Similar experiments were conducted by Sagong and Bobet [11] and Park and Bobet [12], and they also observed the wing-crack at crack tips. In the investigation of crack coalescence pattern of two parallel inclined cracks under compression, Wong and Chau [13] observed both wing-crack and shear crack initiating at crack tips.

Stress intensity factors are a key parameter applied in determining cracked structure stability, and they have been extensively investigated, and accordingly a plenty of research results have been published. For the collinear crack issue, Zhu et al. [14–17] presented the stress intensity factor solution for two collinear cracks inside an infinite plane subjected to biaxial compression by using boundary collocation method and the complex stress function theory, and they proposed a fracture criterion for collinear cracks under compression. Zheng et al. [18] presented the theoretical solution for three collinear cracks inside an infinite plane subjected to biaxial compression. Although theoretical study for collinear crack under compression has been implemented, experimental study and numerical study are also necessary for further understanding the mechanism of collinear crack propagation and interaction and should be implemented.

In this paper, experimental study and numerical study on the fracture property of three collinear cracks under compression are implemented. In the experimental study, the concrete specimens with three collinear cracks are loaded by a true triaxial compression device. In the numerical study, the Abaqus code is employed, and the numerical results agree with the test results.

## 2. Experimental Study

**Figure 1:**
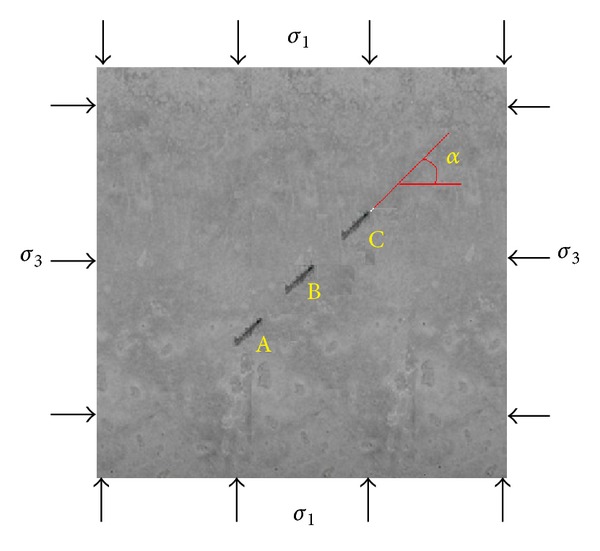
A concrete specimen with three collinear cracks.

The specimens were square plates, 180 mm × 180 mm × 50 mm, with three collinear artificial and penetrated cracks, which measure 20 mm in length, as shown in Figure 1. The material is cement mortar, and the ratio of cement, sand, and water is 1 : 1 : 0.35 by weight. The cracks were made by using a very thin, 0.1 mm, film. The films were placed inside the samples during the process of casting in a mold until they were loaded. The curing period of the samples is 28 days. It is found that, after the specimens have been stored in a heating apparatus with controlled temperature 130°C for 2 hrs, the films can be easily pulled out from the specimens. The crack length and their interval distance are the same and equal 1.0 cm.

**Figure 2:**
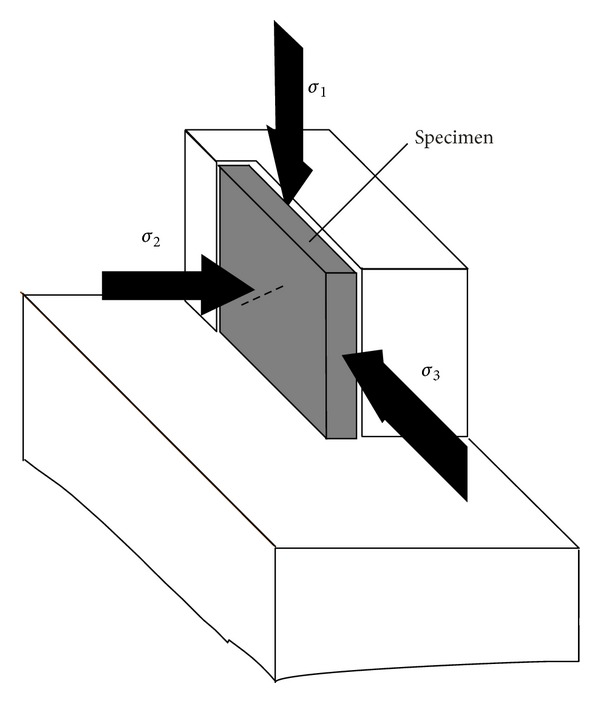
Sketch of a true triaxial loading device and the specimen.

The specimens were loaded by a triaxial loading device; the vertical loading is the major principal stress *σ*_1_, and the two horizontal confining stresses are kept as constants during the process of vertical loading. One of the horizontal stress is *σ*_3_, and the other one is *σ*_2_, as shown in Figure 2. In order to avoid the specimen damage before testing, the specimens, at beginning, are loaded simultaneously along three directions, and the maximum load is selected as the critical load of the specimens.

In order to avoid the effect of the friction between the specimen and the loading device, the specimen surfaces were smeared with oil before testing.

## 3. Effect of Confining Stress

In order to study the effect of confining stress, the specimens are designed to have the same angle 60° between the crack and the* x*-axis. The middle principal stress is kept as a constant *σ*_2_ = 15.0 MPa, and the minor principal stress *σ*_3_ is 0.0 MPa, 2.5 MPa, 5.0 MPa, 7.5 MPa, 10.0 MPa, 12.5 MPa, and 15.0 MPa, respectively.

**Figure 3:**
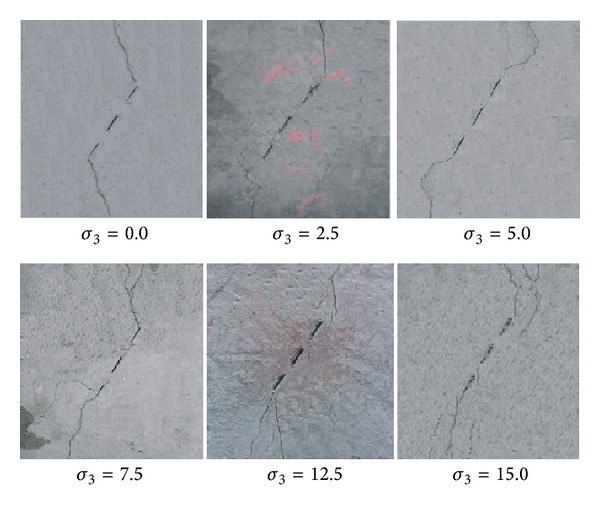
Fracture patterns under different confining stress.

The failure patterns of the specimens under different confining stresses are shown in Figure 3. It can be seen that under low confining stresses, the new cracks deviate in a large angle from the preexisting cracks, whereas, under high confining stress, the new cracks tend to parallel the preexisting crack.

**Figure 4:**
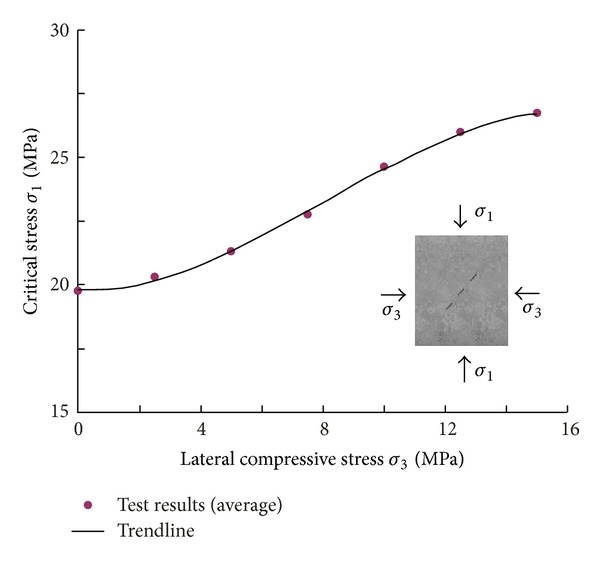
Relation of critical stress *σ*_1_ versus the confining stress *σ*_3_ (*σ*_2_ is kept as a constant).

Figure 4 shows the relationship between the average critical compressive stress *σ*_1_ and the confining stress *σ*_3_. It can be seen that *σ*_1_ increases with *σ*_3_, and the corresponding trend line of this test result is

(1) $$\begin{array}{c} \sigma_{1}=-0.003\sigma_{3}^{3}+0.087\sigma_{3}^{2}-0.04\sigma_{3}+19.8. \end{array}$$

## 4. Effect of Crack Inclination Angles

**Figure 5:**
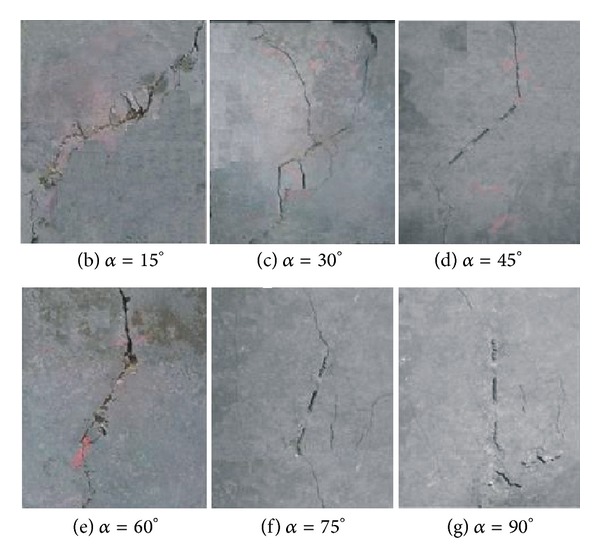
Crack fracture patterns for the specimens with different inclination angles.

**Figure 6:**
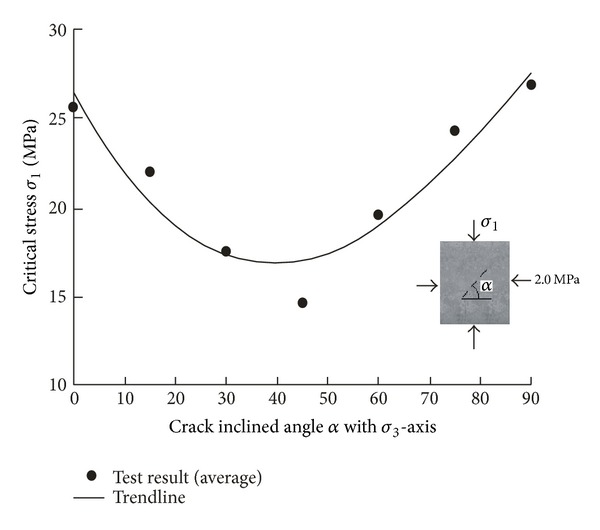
Relation of compressive strength *σ*_1_ with the crack inclination angle *α*.

In order to investigate the effect of crack inclination angles on crack initiation and propagation, the specimens are designed with the following inclination angles: *α* = 0°, 15°, 30°, 45°, 60°, 75°, and 90°. The confining stresses are kept as constants *σ*_2_ = 2.5 MPa and *σ*_3_ = 2 MPa, and we measure the critical vertical stress *σ*_1_. The fracture patterns are shown in Figure 5. It can be seen that the fracture patterns vary with crack inclination angles *α*. As *α* is less than 30°, the fracturing occurs in almost all the crack tips, and as *α* is larger than 75°, several new cracks which are near parallel to the preexisting crack can be observed.  Figure 6 shows the relation of the critical stress *σ*_1_ versus crack inclination angles *α*. One can find that as the crack inclination angle is 45°, the critical stress is the lowest. This is because, under compression, the crack model of inclined cracks belongs to mode II at the beginning, and, as *α* is 45°, the shear stress along the crack surface is the largest; thus, the corresponding stress intensity factor is the largest. The trend line of the test results in Figure 6 is

(2) $$\begin{array}{c} \sigma_{1}=-0.00002\sigma_{3}^{3}+0.082\sigma_{3}^{2}-0.5269\sigma_{3}+26.434. \end{array}$$

## 5. Numerical Study

**Figure 7:**
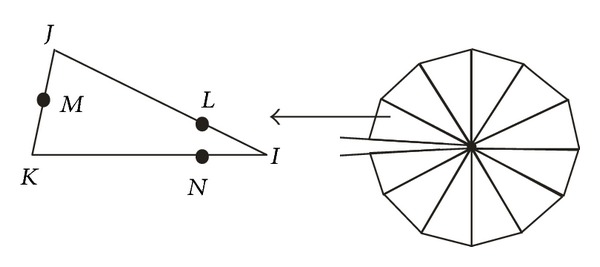
Six node triangular elements around a crack tip.

In order to examine the experimental results, the corresponding numerical study by using Abaqus code is implemented. In this simulation, except for the zone near crack tips where triangular element CPS6 is applied, as shown in Figure 7, quadrilateral element CPS8 is employed. The J-integration method is applied in calculating crack tip stress intensity factor (SIF), and the total elements number is 13782.

**Figure 8:**
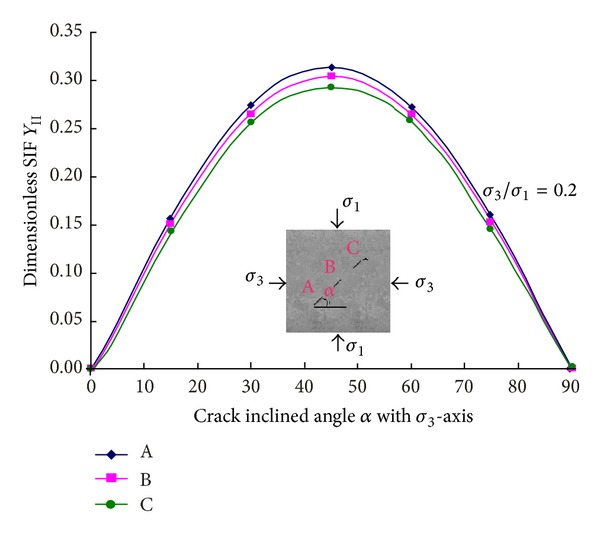
*Y*_*ΙΙ*_ at points A, B, and C from Abaqus.

Figure 8 shows the calculation results for the relation of the dimensionless SIF *Y*_II_ versus crack inclination angles at crack tips A, B, and C, respectively. It can be seen that at crack tip A, the *Y*_II_ value is larger than those at tips B and C, and it is lowest at tip C. However, the maximum difference at tips A and C is less than 7% which is very small, and because tip C is near the boundary which has a significantly effect on crack initiation, and, therefore, the new crack initiated from tip C to the loading boundary, which may spend the minimum energy during propagation, always can be observed in Figure 5.

**Figure 9:**
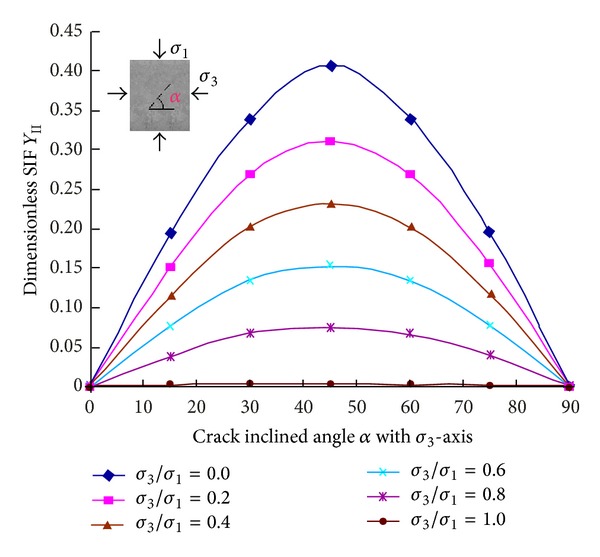
Curves of *Y*_*ΙΙ*_ of crack tip A under different confining stresses and angle.

Figure 9 shows the relation of the SIF of crack tip A versus the ratio of confining stress to the major principal stress *σ*_3_/*σ*_1_, and meanwhile it also shows the relation of the SIF versus crack inclination angles. It can be seen that generally the SIF decreases as the minor principal stress *σ*_3_ (or the ratio *σ*_3_/*σ*_1_) increases, and as *σ*_3_/*σ*_1_ = 1, the SIF values are near zero; the SIF changes with the crack inclination angles, and as the crack inclination is 45°, the SIF is the largest under the same ratio of *σ*_3_/*σ*_1_.

## 6. Conclusion

In this paper, the fracture property of three collinear cracks under compression has been studied by using cement mortar specimens loaded by a true triaxial loading device. Meanwhile numerical study by using Abaqus code has also been implemented, and J-integration method has been applied in calculating crack stress intensity factor. Through the experimental and numerical studies, the following conclusions can be obtained.

1. The critical stress changes with crack inclination angles, and as the crack inclination angle is 45°, the critical stress is the lowest.
2. As crack inclination angle *α* is less than 30°, the fracturing occurs in almost all the crack tips, and as *α* is larger than 75°, several new cracks which are near parallel to the preexisting crack can be observed.
3. The confining stress affects the compressive strength significantly, and as the confining stress increases, the compressive strength increases.
4. Under low confining stress, the new cracks deviate in a large angle from the preexisting cracks, whereas, under high confining stress, the new cracks tend to parallel the preexisting crack.
